# Low Hysteresis Vanadium Dioxide Integrated on Silicon Using Complementary Metal‐Oxide Semiconductor Compatible Oxide Buffer Layer

**DOI:** 10.1002/smsc.202400398

**Published:** 2024-10-30

**Authors:** Swayam Prakash Sahoo, Matthieu Bugnet, Ingrid Cañero Infante, Victor Pierron, Laurence Méchin, Rebecca Cervasio, Pierre Hemme, Jean‐Blaise Brubach, Pascale Roy, Luc G. Fréchette, Anne D. Lamirand, Bertrand Vilquin

**Affiliations:** ^1^ Ecole Centrale Lyon INSA Lyon Université Claude Bernard Lyon 1 CNRS Institut des Nanotechnologies de Lyon (INL) UMR 5270 69130 Ecully France; ^2^ Laboratoire Nanotechnologies Nanosystèmes (LN2) ‐ CNRS UMI‐3463 Université de Sherbrooke Sherbrooke QC J1K 0A5 Canada; ^3^ Institut Interdisciplinaire d’Innovation Technologique (3IT) Université de Sherbrooke Sherbrooke QC J1K 0A5 Canada; ^4^ CNRS INSA Lyon Université Claude Bernard Lyon 1 MATEIS, UMR 5510 69621 Villeurbanne France; ^5^ CNRS INSA Lyon Ecole Centrale de Lyon Université Claude Bernard Lyon 1 CPE Lyon INL UMR 5270 69621 Villeurbanne France; ^6^ Normandie Univ UNICAEN ENSICAEN CNRS GREYC (UMR 6072) 14000 Caen France; ^7^ AILES Beamline Synchrotron SOLEIL ‐ CNRS ‐ CEA Paris‐Saclay L’Orme des Merisiers 91192 Gif‐sur‐Yvette Cedex France

**Keywords:** M1–M2 structural phase transition, metal‐insulator phase transitions, Mott‐Peierls transition, strain‐influenced hystereses, Vanadium dioxide

## Abstract

VO_2_ undergoes a metal‐insulator transition (MIT) at ≈70 °C, which induces large variations in its electrical and wavelength‐dependent optical properties. These features make VO_2_ a highly sought‐after compound for optical, thermal, and neuromorphic applications. To foster the development of VO_2_‐based devices for the microelectronic industry, it is also imperative to integrate VO_2_ on silicon. However, high lattice mismatch and the formation of silicates at the interface between VO_2_ and Si degrade the quality and functionality of VO_2_ films. Moreover, VO_2_'s polymorphic nature and stable V—O phases pose integration issues. To address these challenges, the MIT of VO_2_ thin films integrated on Si with a complementary metal‐oxide semiconductor‐compatible Hf_*x*_Zr_1−*x*_O_2_ (HZO) buffer layer is investigated. Using in situ high‐resolution X‐ray diffraction and synchrotron far‐infrared spectroscopy, combined with multiscale atomic and electronic structure characterizations, it is demonstrated that VO_2_ on the HZO buffer layer exhibits an unusually low thermal hysteresis of ≈4 °C. In these results, the influence of strain on M2 phase nucleation, which controls the hysteresis, is unraveled. Notably, the rate of phase transition is symmetric and does not change for the heating and cooling cycles, implying no incorporation of defects during cycling, and highlighting the potential of an HZO buffer layer for reliable operation of VO_2_‐based devices.

## Graphical abstract

Strain disrupts the first‐order M1—R phase transition in VO_2_, resulting in an M1—M2—R transition. This study not only underlines the potential of an Hf_*x*_Zr_1−*x*_O_2_ buffer layer for reliable VO_2_‐based devices but also correlates strain and hysteresis width to M2 phase. This analysis is enabled by complementary postmortem and in situ structural, electrical, and optical characterizations.

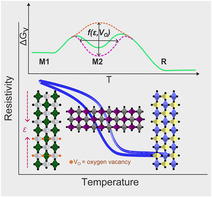

## 1 Introduction

Vanadium dioxide (VO_2_) is a strongly correlated electron system^[^ ^1^ ^]^ with a metal‐insulator transition (MIT) at ≈70 °C^[^ ^2^ ^]^ and has promising potential applications such as in Mott neuromorphic applications as artificial synapses and neurons,^[^ ^3^, ^4^ ^]^ thermotronics,^[^ ^5^ ^]^ optical switches,^[^ ^6^ ^]^ and meta‐holography.^[^ ^7^ ^]^ This MIT accompanies a structural phase transition from room‐temperature monoclinic M1 to elevated temperature tetragonal (rutile, R) structure. While a rich history of work exists on VO_2_, most studies have been carried out on epitaxially grown films on sapphire substrate^[^ ^8^, ^9^ ^]^ to investigate this correlated electron system. Experiments to deposit VO_2_ on silicon with a TiO_2_^[^ ^10^, ^11^, ^12^ ^]^ buffer layer have also been performed as TiO_2_ and metallic VO_2_ can share the same rutile structure (P4_2_/mnm)^[^ ^13^, ^14^ ^]^ with a low lattice mismatch.^[^ ^10^ ^]^ However, VO_2_ deposited on TiO_2_ buffer layers has been reported to generate microcracks.^[^ ^15^, ^16^, ^17^, ^18^ ^]^ To foster the implementation of VO_2_‐based devices, it is crucial to integrate VO_2_ on silicon platform without generating new defects for reliable device operations. Nevertheless, the high lattice mismatch and the formation of oxides and silicates at the interface between VO_2_ and crystalline Si degrade the quality and functionality of the VO_2_ film. Additionally, VO_2_ (M1) is a challenging material to integrate into patterned heterostructures because it can exist as multiple polymorphs (A, B, M1) and the high‐temperature depositions can also lead to the formation of various stable oxides in the V—O system (V_*n*_O_2*n*−1_, V_*n*_O_2*n*+1_), thereby limiting the growth to a tiny window of control on temperature and pressure. In this work, we demonstrate a successful and reproducible growth of high‐quality VO_2_ (M1) film on n^+^Si(100) using crystalline (Hf, Zr)O_2_ (HZO) buffer layer. There are multiple reasons for using HZO. First, it is complementary metal‐oxide semiconductor (CMOS) compatible; second, it has similar monoclinic structure (P2_1_/c)^[^ ^19^ ^]^ as low‐temperature VO_2_; and third, it has similar lattice parameters as Al_2_O_3_ on which VO_2_ has exceptionally low strain in its metallic state.^[^ ^20^ ^]^ Therefore, HZO could impose low‐tensile in‐plane thermal strain on VO_2_ phases across the MIT providing low stress and lower probability of cracking during phase transition. HZO is also transparent in a wide‐infrared (IR) range allowing low‐loss medium for VO_2_‐based devices.^[^ ^21^, ^22^ ^]^ Lastly, the high dielectric constant^[^ ^23^ ^]^ (20 < *ε*_r_ < 35) and low thermal conductivity^[^ ^24^ ^]^ (≈1.1 W m^−1^ K^−1^) of crystalline HZO will reduce the leakage current as well as provide a thermal barrier for VO_2_‐based devices on silicon. A recent study^[^ ^25^ ^]^ reported the increase in visible transmittance of VO_2_ films with the introduction of HZO buffer layer on glass substrate. However, the impact of the HZO buffer layer on the MIT characteristics of VO_2_ remains largely unexamined.

We show that using HZO has not only prevented further nucleation of new defects across the MIT but has also reduced the hysteresis width to ≈4 °C. We also present evidences of M2 phase formation during the MIT, which directly affects its hysteresis width. This is confirmed by a combination of postmortem and in situ structural, electrical, and optical characterization performed across the MIT.

## 2 Results and Discussion

In this study, five samples were investigated. They correspond to VO_2_ deposition, detailed in Experimental Section, at 700 °C on four different substrates: *c–*sapphire, n^+^Si(100), n^+^Si(100) substrate with either a monoclinic or tetragonal HZO buffer layer, and n^+^Si(100) substrate with a tetragonal HZO buffer layer at 620 °C. These samples will be referred to as Al_2_O_3_/VO_2_, Si/VO_2_, HZO[m]/VO_2_, HZO[t]/VO_2_, and 620_HZO[t]/VO_2_, respectively, throughout the manuscript.

**Figure 1:**
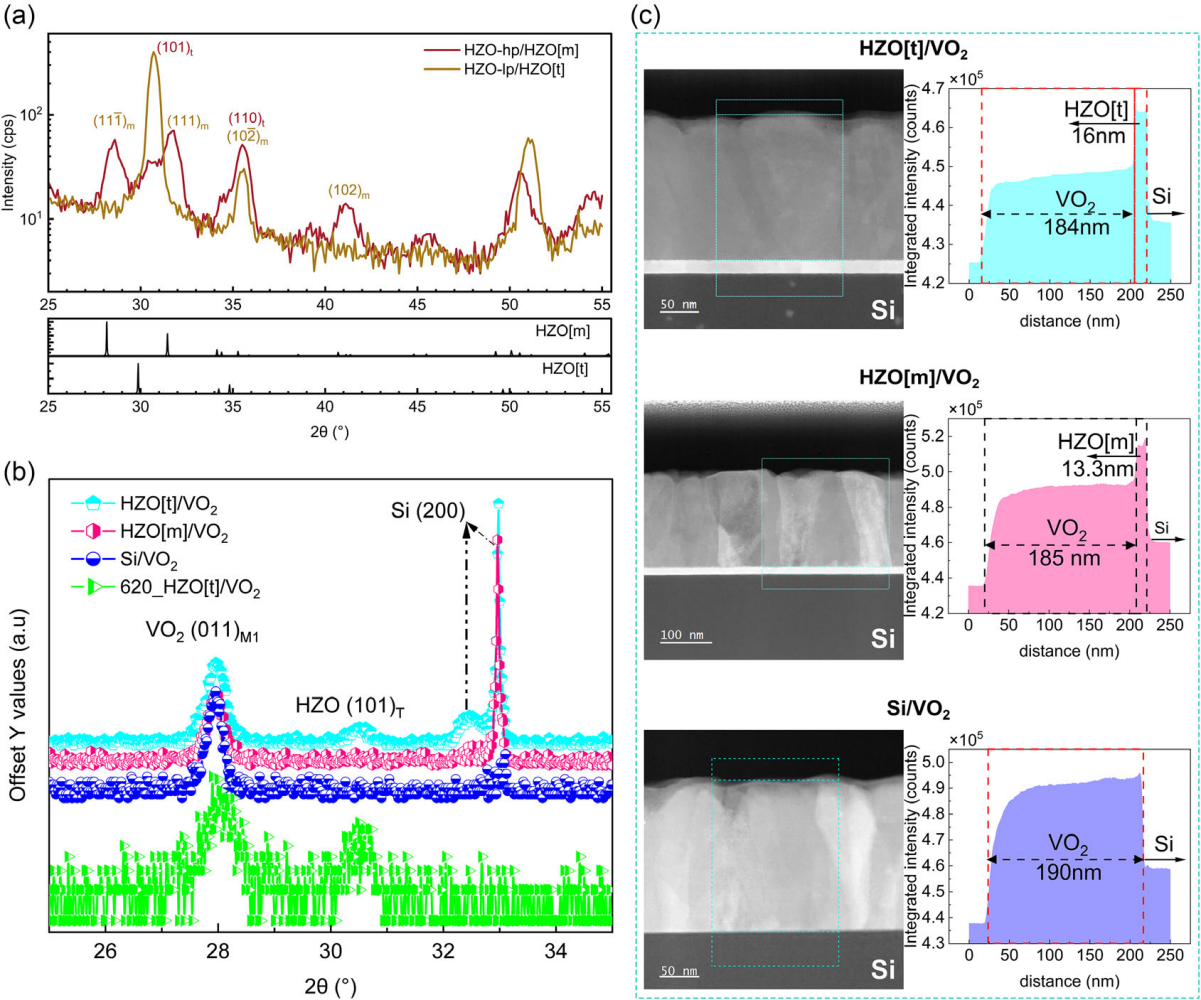
Structural characterization. a) GIXRD diffractogram on 16 nm HZO‐lp(HZO[t]) and 13.3 nm HZO‐hp(HZO[m]) buffer layers. The crystallographic phase of HZO is shown to be controlled by the working pressure. b) Specular (*θ*/2*θ*) high‐resolution XRD (HRXRD) scan between 25° and 30° illustrating (011) out‐of‐plane orientation of VO_2_. c) Low‐magnification HAADF image of samples showing thicknesses of VO_2_ and HZO (t/m).

The crystallographic phase of HZO buffer film is influenced by the working pressure during the deposition. To characterize its effect on VO_2_, HZO was deposited at high pressure (5 × 10^−2^ mbar, HZO‐hp) and at low pressure (5 × 10^−3^ mbar, HZO‐lp). The grazing incidence X‐ray diffraction (GIXRD) shown in **Figure** **1**a illustrates with ($1\bar{1}1$)_m_ and (111)_m_ reflections that post‐annealed HZO‐hp crystallizes predominantly in monoclinic phase (P2_1_/c) (ICDD PDF card no. 04‐006‐7678 and JCPDS 37‐1484^[^ ^26^ ^]^) and HZO‐lp crystallizes exclusively in tetragonal phase (P4_2_/nmc) with (101)_t_ reflection.^[^ ^27^ ^]^ The differing phases could arise from the fact that the low‐pressure deposition leads to a Zr‐rich phase compared to the high‐pressure deposition.^[^ ^28^ ^]^ The XRR fitting results (see Figure S1a, Supporting Information) illustrate the effect of pressure on the surface roughness of annealed HZO. The HZO‐lp has a lower surface roughness compared to the high‐pressure deposition (*σ*_rms_ = 0.5 nm vs *σ*_rms_ = 1.1 nm). The faster decay of Kiessig fringes in the case of HZO‐hp depicts a rougher interface with Si in comparison to the abrupt interface of HZO‐lp, which is confirmed by scanning transmission electron microscopy in high‐angle annular dark‐field (STEM–HAADF) imaging conditions in Figure 1c. The thickness of HZO‐lp (hereafter, HZO[t]) and HZO‐hp (hereafter, HZO[m]) are 16 and 13.3 nm, respectively, as confirmed by the low‐magnification STEM–HAADF images in Figure 1c) and the modeling of XRR results.

Specular (*θ*/2*θ*) high‐resolution XRD (HRXRD) of VO_2_ deposited on bare silicon as well as on HZO were performed between 15° and 75° (see Figure S1b, Supporting Information). Figure S1b, Supporting Information, also includes VO_2_ deposited on Al_2_O_3_(0001) where VO_2_ grows epitaxially with (002) planes parallel to the substrate similar to observations in literature.^[^ ^29^ ^]^ Furthermore, the HRXRD plot indicates the absence of other polymorphs and ordered oxides in all samples. Figure 1b shows the magnified region of this HRXRD between 25° and 35°. VO_2_ crystallizes in its monoclinic phase (ICDD PDF card no. 04‐003‐2035) in all samples (HZO[m], HZO[t] and Si), with (011) planes oriented parallel to the substrate. The thicknesses of VO_2_ films in this study were targeted to be 183 ± 2 nm, which is confirmed experimentally using STEM–HAADF imaging (see Figure 1c), despite a roughness of several nanometers. Si(200)‐forbidden peaks^[^ ^30^ ^]^ occur at ≈32.98° with shoulders at ≈32.5°. The peak at 30.5° belongs to the (101) plane of tetragonal HZO.

**Figure 2:**
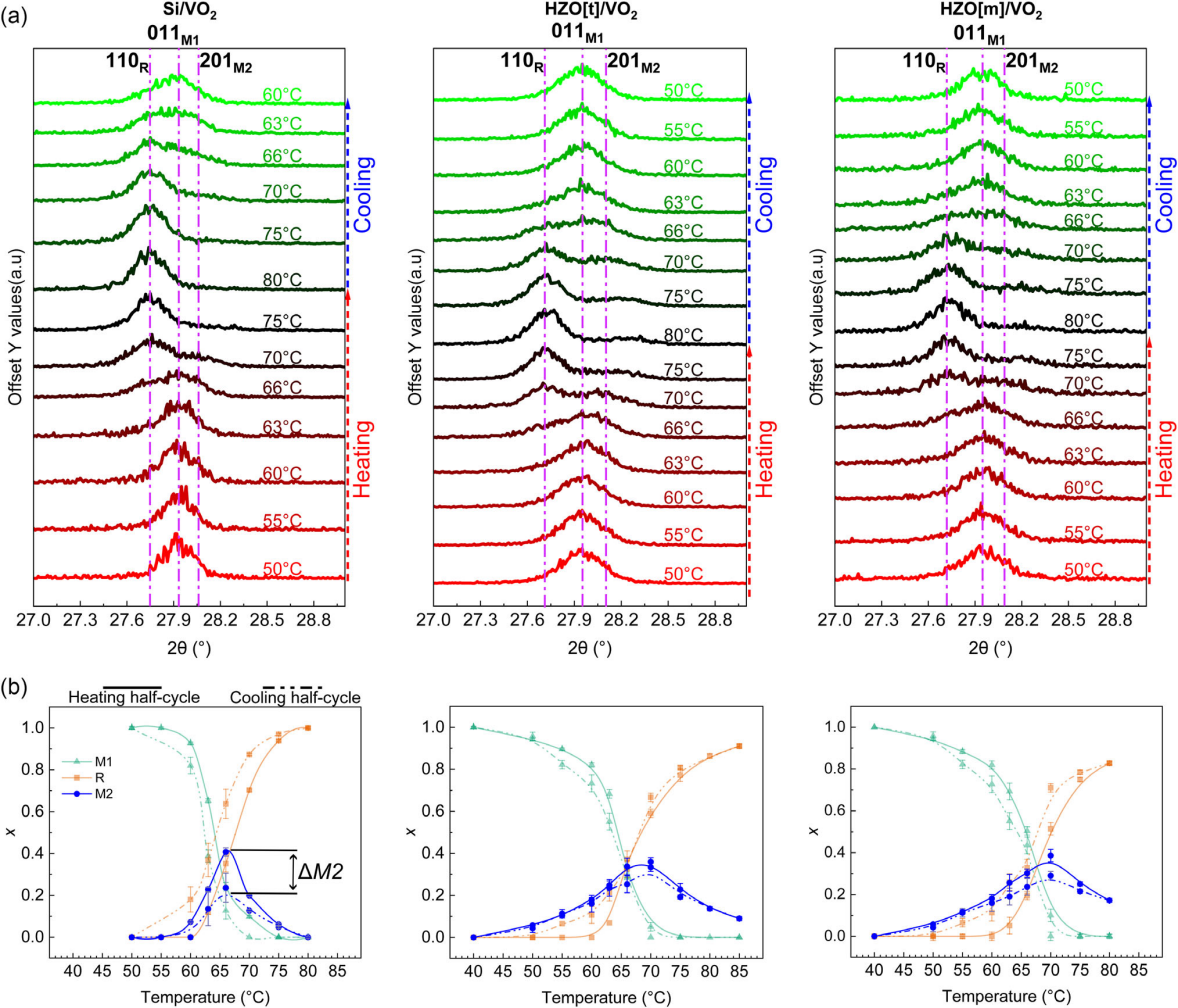
a) High‐resolution XRD performed at various temperatures across the metal‐insulator transition temperature of VO_2_ for three different samples. b) The corresponding graphs illustrate the evolution of the volume fraction of M1, R, and M2 phases with temperature for these samples.

Temperature‐dependent *θ*/2*θ* HRXRD scans were performed on the different samples, as illustrated in **Figure** **2**a. The phase transition of VO_2_ from monoclinic (M1) to tetragonal rutile (R) (P4_2_/mnm) structure occurs with (011)_M1_ plane at ≈27.93° transforming into (110)_R_ (ICDD PDF card no. 01‐079‐1655) at ≈27.75°. This structural transition generates tensile strains along [011]_M1_. We also observe the formation of the VO_2_ M2 phase (ICDD PDF card no. 00‐033‐1441) at ≈28.07°. The strain‐induced M2 phase deviates MIT from the first‐order phase transition^[^ ^31^ ^]^ and has been reported to slow down the switching to the metallic rutile phase and shifts the transition toward higher temperature. Figure 2b illustrates the evolution of the phase fraction of M1, M2, and R with temperature for each of these samples. The change in M2 phase (ΔM2) at the *T*_MIT_ can be seen to be largest for Si/VO_2_ and is minimum for HZO[t]/VO_2_. In our studies, the M2 phase does not occur as a twinned structure as it has been previously reported.^[^ ^31^, ^32^ ^]^ Previous works on various VO_2_ samples also do not report evidence of twinning in the M2 phase.^[^ ^33^, ^34^, ^35^ ^]^ VO_2_ is deposited in its tetragonal phase due to elevated temperature deposition and cools down to room‐temperature monoclinic phase. During this cooling process, strain is induced by the phase transition. In all the samples, we hypothesize that the strain rate on VO_2_ is lower than the critical threshold for inducing twinning of the M2 phase.

**Figure 3:**
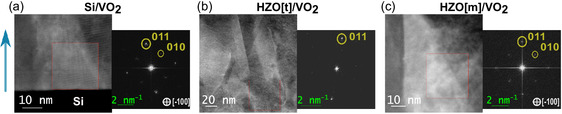
STEM–HAADF and FFT analysis of a) Si/VO_2_, b) HZO[t]/VO_2_, and c) HZO[m]/VO_2_. The blue arrow indicates the growth direction of VO_2_ for all samples.

The microstructure of the samples was investigated at room temperature at the nanoscale using STEM–HAADF imaging. STEM–HAADF of each sample is shown in **Figure** **3**, along with fast Fourier transform (FFT) for phase identification. The columnar growth of grains is observed for each specimen, as shown in Figure 1c, with a single grain growing from the substrate (HZO or Si) up to the top of the film. The non‐equiaxed microstructure in VO_2_ film suggests that the deposition took place at a low relative temperature (TsTm<0.5; for our case, it is 7001967=0.36) to inhibit substantial grain‐boundary (GB) movement during coalescence or thickening. As the film thickness increases, grain size increases due to faster‐growing grains overshadowing the slower‐growing ones, both within and perpendicular to the plane of the film. This behavior typically occurs in materials and systems characterized by significant growth velocity anisotropies, low surface self‐diffusivities, and limited GB mobilities.^[^ ^36^ ^]^ The FFT patterns extracted from high‐resolution images provide average values for *d*_hkl_. The *d*_hkl_ values of various planes along with their strain with respect to bulk *d–*spacings are also tabulated in **Table** **1**.

**Table 1:** *d*_hkl_ of VO_2_ derived from FFT in Figure 3.

Samplea)	Interplanar spacings [Å]			
	*d*_011_	*d*_101_	*d*_010_	*d*_001_
Si/VO_2_	3.16 (−1.44%)	3.17 (−1.31%)	4.51 (−6.86%)	4.99 (+3.06%)
HZO[t]/VO_2_	3.22 (+0.35%)	–	–	–
HZO[m]/VO_2_	3.21 (+0.14%)	3.15 (−1.65%)	4.48 (−7.59%)	4.52 (−6.65%)
VO_2_ bulk [REF: ICDD PDF card no. 04‐003‐2035]	3.206720		4.843040	

a) Within the parenthesis is the magnitude of strain with respect to bulk *d*–spacing. ± signs mean tension and compression, respectively.

The out‐of‐plane *d*_011_ of VO_2_ in sample Si/VO_2_ is in compressive state (≈−1.44%) with respect to the bulk. However, *d*_011_ in HZO[t]/VO_2_ and HZO[m]/VO_2_ are in tensile state (≈+0.35% and +0.14%, respectively). Consequently, the in‐plane compression of *d*_101_ in samples Si/VO_2_ and HZO[m]/VO_2_ are ≈−1.31% and ≈−1.65%, respectively. Although strain values on sample HZO[t]/VO_2_ could not be retrieved, they are expected to be lower compared to monoclinic HZO buffer layer because of the larger out‐of‐plane stress exerted by tetragonal HZO, which leads to even smaller in‐plane compression owing to Poisson effect.

**Figure 4:**
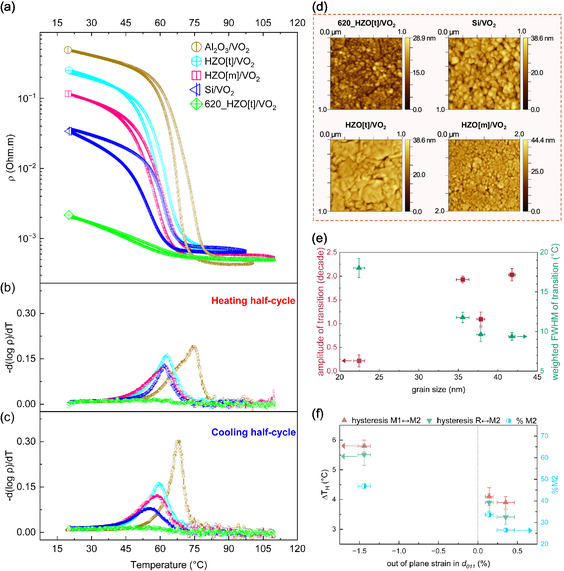
a) Thermal hysteresis loops of VO_2_. b,c) Derivative of the resistivity curve ($\frac{-\partial \log\rho }{\partial T}$) among different samples for the heating and cooling regime of the cycle. d) AFM topography of VO_2_ thin films on silicon platform. e) Influence of grain size on the amplitude and rate (FWHM of $\frac{-\partial \log\rho }{\partial T}$) of phase transition. f) Influence of out‐of‐plane strain in d_011_ on hysteresis width, Δ*T*_H_ and %M2 phase. (e,f) The bars indicate the measurement errors.

**Figure** **4** shows the cyclic measurement of the electrical resistance of VO_2_ as a function of temperature across its MIT for different samples. The discussion on MIT pivots^[^ ^37^ ^]^ around the following: 1) the transition temperatures, namely *T*_h_ and *T*_c_ defined as the maxima of $\frac{-\partial \log\rho }{\partial T}$ within the heating and cooling regime of the cycle; 2) their full width at half maximum (FWHM), which provides insights into the defect content and consequently the rate of the phase transition; and 3) the amplitude of the transition defined as the area under the derivative curve, which informs about the magnitude of the separation between the resistances of the two phases.^[^ ^37^, ^38^ ^]^

The room‐temperature resistivity of all films in their insulating state is shown in Figure 4a. To unravel the effect of grain size with other properties and directly compare our data with literature, VO_2_ films were grown on HZO[t] at 620 °C. Strong differences in room‐temperature conductivity are observed. It has been demonstrated in the literature that GBs present much larger conductivity than that of grain core in the insulating phase of VO_2_ film, probably as GBs with unpaired V atoms are more energetically favorable than GBs with V—V dimerization.^[^ ^39^ ^]^ Moreover, GBs introduce electron trapping at gap states in insulating phase and electron scattering in R phase and provide ideal sites for the agglomeration of oxygen vacancies as their nucleation is favorable there.^[^ ^40^ ^]^ Oxygen vacancies in VO_2_ are known to stabilize the metallic state at lower temperature than stoichiometric VO_2_ and decrease the resistivity of the insulating phase resulting in a flattening and shifting of the hysteresis.^[^ ^41^, ^42^ ^]^ Two mechanisms are considered to explain this behavior. First, oxygen vacancies are, with interstitial defects, native point defects and act as electron donors in the VO_2_ lattice. Second, they destabilize the V—V dimer in M1 phase, which shrinks the Peierls gap separating the bonding $\left(d_{\left|\right|}\right)$ and the anti‐bonding level $\left(d_{\left|\right|}^{*}\right)$ of the V—V dimer^[^ ^43^, ^44^, ^45^ ^]^ and raises the Fermi level (*E*_F_) closer to the conduction band. Therefore, the decrease of the transition temperature in our samples (≈15 °C, data provided in Table S1, Supporting Information), which is evidenced in Figure 4b,c, is due to the combined impact of strain (discussed later) as well as the presence of oxygen vacancies in all samples, giving rise to a deviation of about 2% maximum. Figure 4e reveals the effect of grain size and consequently of the density of GBs, measured from topographic atomic force microscopy (AFM) images as shown in Figure 4d, on the MIT characteristics in these films. It is observed that sample HZO[t]/VO_2_ has the largest grain size and its FWHM of transition is around 9.5 °C. Larger grains (hence, lower density of GBs) provide a lower density of defects within the bandgap which reduces the weighted FWHM and enhances the amplitude of MIT as reported before.^[^ ^38^ ^]^ Additionally, the difference in the resistances in the metallic state is attributed to the delayed relaxation of carriers (defect‐induced band tailing).^[^ ^46^ ^]^ The smaller grains have higher defect concentration, which diminishes the electronic resistivity.

The thermal hysteresis width Δ*T*_H_ is defined by *T*_h_ − *T*_c_. In our case, the results of the peak fitting (see Figure S2, Supporting Information) of the $\frac{-\partial \log\rho }{\partial T}$ curves reveal two distinct maxima for each of the heating and cooling half‐cycle. These peaks could be attributed to the M1—M2 and M2—R transitions, whose evolutions are in good agreement with those observed in XRD (T) measurements. Similar interpretations of IR transmittance experiments^[^ ^47^ ^]^ have been reported before. Specifically, it was evidenced on VO_2_ microbeams^[^ ^31^ ^]^ that compressive strain leads to the M1—R transition, and tensile strain leads to the M1—M2—R transition. Although there exists compressive strain along *d*_011_ in Si/VO_2_ and tensile strain on samples with HZO buffer layer, our samples follow the M1—M2—R route. Previous works^[^ ^48^, ^49^, ^50^, ^51^ ^]^ have revealed that the proportion of M2 phase is much higher on *c–*sapphire as compared to Si(100) due to larger biaxial in‐plane strain in the direction perpendicular to the [100] (*c*_R_ axis). We also observe higher proportion of the M2 phase for VO_2_ grown on *c–*sapphire. This is qualitatively observed in Figure S2, Supporting Information (and, quantitatively in Table S1, Supporting Information) where the deconvolution of the area under the curve of $\frac{-\partial \log\rho }{\partial T}$ that corresponds to M2 phase can be compared for VO_2_ on different samples. The quantitative data extracted from the hysteresis and amplitude of the transition observed in the samples studied here are compiled in Table S1, Supporting Information.

The macroscopic electric resistance depends on the percolation of the metal domains forming an electrically conductive path. This in turn is dependent on the nucleation probability of the rutile domains and the kinetics of their growth and coalescence leading to percolation.^[^ ^52^ ^]^ The phase transition route M1—M2—R involving the nucleation of the M2 phase has been suggested^[^ ^20^ ^]^ to contribute to an increase in hysteresis width. Our results are congruent with this claim as high out‐of‐plane compressive (in‐plane tensile) strain along *d*_011_(VO_2_[M1]) stabilizes the M2 phase and consequently leads to an increase in hysteresis, as shown in Figure 4f.

The thermodynamic driving force for phase transition is defined^[^ ^37^, ^53^ ^]^ by Δμ=−2γrc, where *γ* is the interfacial energy, and *r*_c_ is the critical nucleus. The fitting procedure effectively captures both the nucleation and growth processes, as it accounts for the phase fraction of any phase (M1, M2, or R) transitioning from 0 to 1. The rationale behind our preference for using the nucleation rate as the rate determining step is detailed as follows: in all samples, VO_2_ grains extend from the interface with the substrate to the top of the film (see Figure 1c). As the sample is heated from beneath the substrate, it is assumed that the nucleation of the M2 phase initiates at the interface between VO_2_ and either Si or HZO and progresses upward, following the natural heat gradient—a phenomenon corroborated by observations published in literature.^[^ ^54^ ^]^ After nucleation, it is assumed that the growth velocity of the first‐order diffusionless M2—R phase transition remains consistent across similarly textured samples. This first approximation simplifies the analysis by allowing us to treat M1—M2 nucleation as a thermodynamic barrier, even though it does not account for the effects of defects and intrinsic strain on heat diffusion, which could cause variations in growth rates. A comparable evolution of thermodynamic potential with the radius of nuclei during a first‐order phase transition, driven by the curvature dependence of surface tension (as described by the Gibbs–Thomson effect), has been theoretically proposed by Ulbricht et al.^[^ ^55^ ^]^ providing insights into how strain influences the nucleation of M2 phase embryos. The results of the peak fitting of $\frac{-\partial \log\rho }{\partial T}$ shown in Figure S2, Supporting Information, reveal that the percentage of total domains undergoing M1—M2 transition in each half‐cycle (%M2 phase formation) is highest on *c–*sapphire and reduces on Si, HZO[m] and HZO[t]. This is due to the in‐plane strain energy experienced by VO_2_ tetragonal phase which scales in the order: γ_sapphire_ > γ_Si_ > γ_HZO[m]_ ∼ γ_HZO[t]_. The origin of this strain could be the different thermal expansions of the underlying layer (HZO, Si, and *c–*sapphire) of VO_2_, which offer different in‐plane stress during the post‐growth cooling process. In the case of Si/VO_2_, the MIT occurs faster (smaller FWHM) in comparison to HZO[t]/VO_2_ during the heating half‐cycle, which is due to compressive strain as discussed earlier. But during the cooling cycle, the nucleation for phase transition takes longer in Si/VO_2_, implying that the critical size of nucleus has been reduced because the film has incorporated more defects during the transition. This incorporation of defects leads to a sluggish transition, i.e., increased FWHM of the $\frac{-\partial \log\rho }{\partial T}$, which is illustrated by an increased hysteresis. Samples HZO[t]/VO_2_ and HZO[m]/VO_2_ show the symmetric nature of $\frac{-\partial \log\rho }{\partial T}$, which suggests the symmetries of not only the probability of nucleation and growth of metallic domains but also the generation and release of strain during the cyclic ρ–T measurements. Since hysteresis quantifies^[^ ^56^ ^]^ the dissipation of internal energy by creation of defects which subsequently would lead to crack formation, the lower hysteresis widths observed in HZO[t]/VO_2_ and HZO[m]/VO_2_ highlights the impact of HZO buffer layer in hindering new defect generation. The significantly lower amplitude of resistivity and large FWHM of $\frac{-\partial \log\rho }{\partial T}$ for 620_HZO[t]/VO_2_ stems from lower crystallinity of VO_2_, due to its lower thickness and low deposition temperature. HZO thus acts as an optimum buffer layer in lowering the strain and subsequently the formation of M2 and reduces the hysteresis compared to *c*–sapphire.

Narrower hysteresis width in VO_2_ is desirable in many applications. It has been experimentally demonstrated^[^ ^57^ ^]^ that a narrower hysteresis width allows low programming voltages to perform resistive switching in VO_2_, thereby, consuming less energy. Applications such as electric pass polarizer^[^ ^58^ ^]^ can reduce its power consumption and nonvolatile optical memory^[^ ^59^, ^60^ ^]^ can be realized by leveraging the narrower hysteresis. A narrower hysteresis is also vital for sensors applications^[^ ^37^, ^61^ ^]^ that demand consistent and reliable behavior with minimal lag when the temperature fluctuates between heating and cooling cycles leading to better performance, through precise temperature sensing and control.

**Figure 5:**
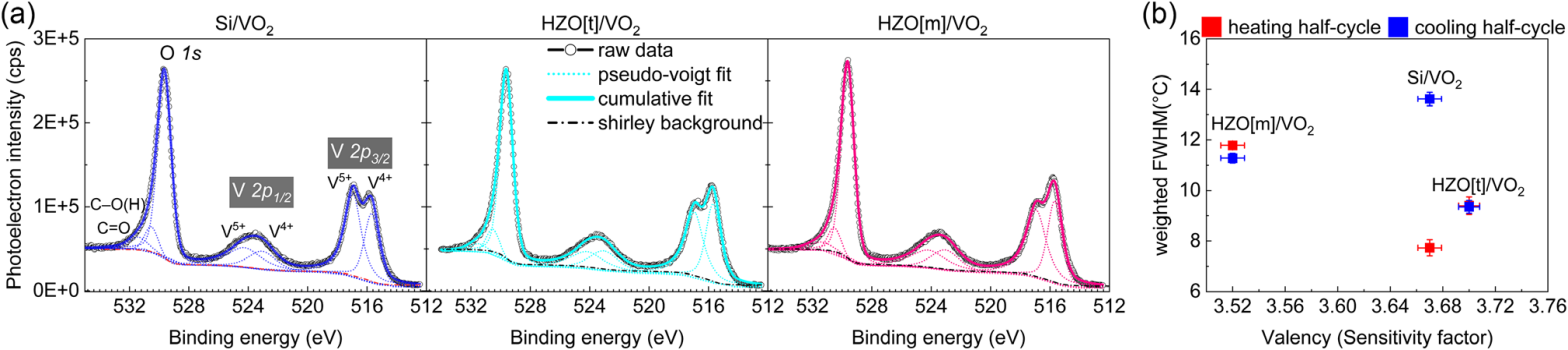
a) Core‐level XPS spectra of vanadium (V 2*p*) and oxygen (O 1*s*). b) Influence of oxygen content on the rate of phase transition, by the representation of the weighted FWHM of MIT as function of the V_2_O_*x*_ valency inferred from XPS core levels with sensitivity factors method.

**Figure** **5**a provides the core‐level emission spectra of VO_2_ determined by X‐ray photoelectron spectroscopy (XPS) for all samples. The O 1*s* peak is found at 529.6 eV (±0.04 eV), which corresponds to the binding energy of the O—V bonds and does not shift for any growth conditions of VO_2_. As a C 1*s* signal presents two shoulders at 1.5 and 4 eV of C—C binding energy (not shown here), the peak at 530.5 eV is ascribed to C—O(H) and the peak at 531.5 eV is ascribed to other adsorbed oxygen such as C=O.^[^ ^62^ ^]^ V 2*p*_3/2_ peaks present two contributions attributed to V^4+^ for the peak at 515.7 eV (±0.05 eV) and to V^5+^ for the peak at 517 eV (±0.05 eV), in agreement with the literature.^[^ ^63^ ^]^ The separation between the binding energies of V 2*p*_1/2_ and V 2*p*_3/2_ was determined at 7.27 eV for V^5+^ and at 7.36 eV for V^4+^. The clear presence of V^5+^ peak indicates an overoxidation of the film. The global valency, as documented in the literature,^[^ ^64^ ^]^ can be determined through two distinct approaches: first, by calculating the area normalized by sensitivity factors, and secondly, by employing the weight percent average of V 2*p*_3/2_ peaks. The second method addresses a consistent underestimation of the oxidation rate observed in the first one. The first method yields valency values below 4+, which is incongruous since no V 2*p* peak with a valency lower than 4 is detected. This underestimation is ascribed to the considerable variability of sensitivity factors with chemical state and the challenge of determining an appropriate Shirley background. Further reasoning behind our choice of utilizing valency calculated from sensitivity factors is available in Supporting Information.

The high deposition temperature, the features of the MIT in our samples and the high relative reflectance in room‐temperature IR spectroscopy measurements of VO_2_ in Figure S4, Supporting Information clearly attest of the oxygen‐deficient nature of VO_2_. An explanation for the apparent discrepancy between oxygen‐deficient VO_2_ and V^5+^ peaks in XPS would be the enhanced surface oxidation of the films and thus the formation of V_2_O_5_, and the sensitivity of the XPS experiments to the top surface of the films (here, ≈5 nm).

The effect of oxygen content at the surface is shown in Figure 5b. For samples deposited on HZO buffer layer, which have similar order of strain on VO_2_ (+0.5%/0.6%, see Table 1), the FWHM is observed to increase with the excess of oxygen vacancies due to a larger nucleation barrier for the M2 phase in the complete film (i.e., ΔM2 is larger for HZO[m]/VO_2_ than for HZO[t]/VO_2_). The FWHM for HZO[t]/VO_2_ is nearly the same for their respective heating and cooling half‐cycles, thus implying no new incorporation of defects, while for HZO[m]/VO_2_, a minor change is observed. This change is within the resolution of measurement and needs to be investigated further to better understand the defect generation and their propagation during cyclic phase transformations for reliable operation of devices. In the case of Si/VO_2_, the incorporation of defects occurs during the phase transition, which increases the thermodynamic barrier to phase transition and leads to a significant change in the FWHM of transition from 7.7 °C in heating half‐cycle to 13.6 °C in the cooling cycle. This is also hypothesized by the fact that ΔM2 (refer to Figure 2b) is highest for Si/VO_2_ implying that during the cooling half‐cycle, the nucleation of the M2 phase was significantly reduced due to release of strain through defect incorporation. To determine the importance of the defect's incorporation on the phase nucleation, the influence of strain and oxygen content on the nucleation need to be disentangled. Techniques to quantify the oxygen content in the bulk of the film should be employed to confirm our suggestion regarding the influence of oxygen content on the nucleation of the M2 phase in VO_2_.

**Figure 6:**
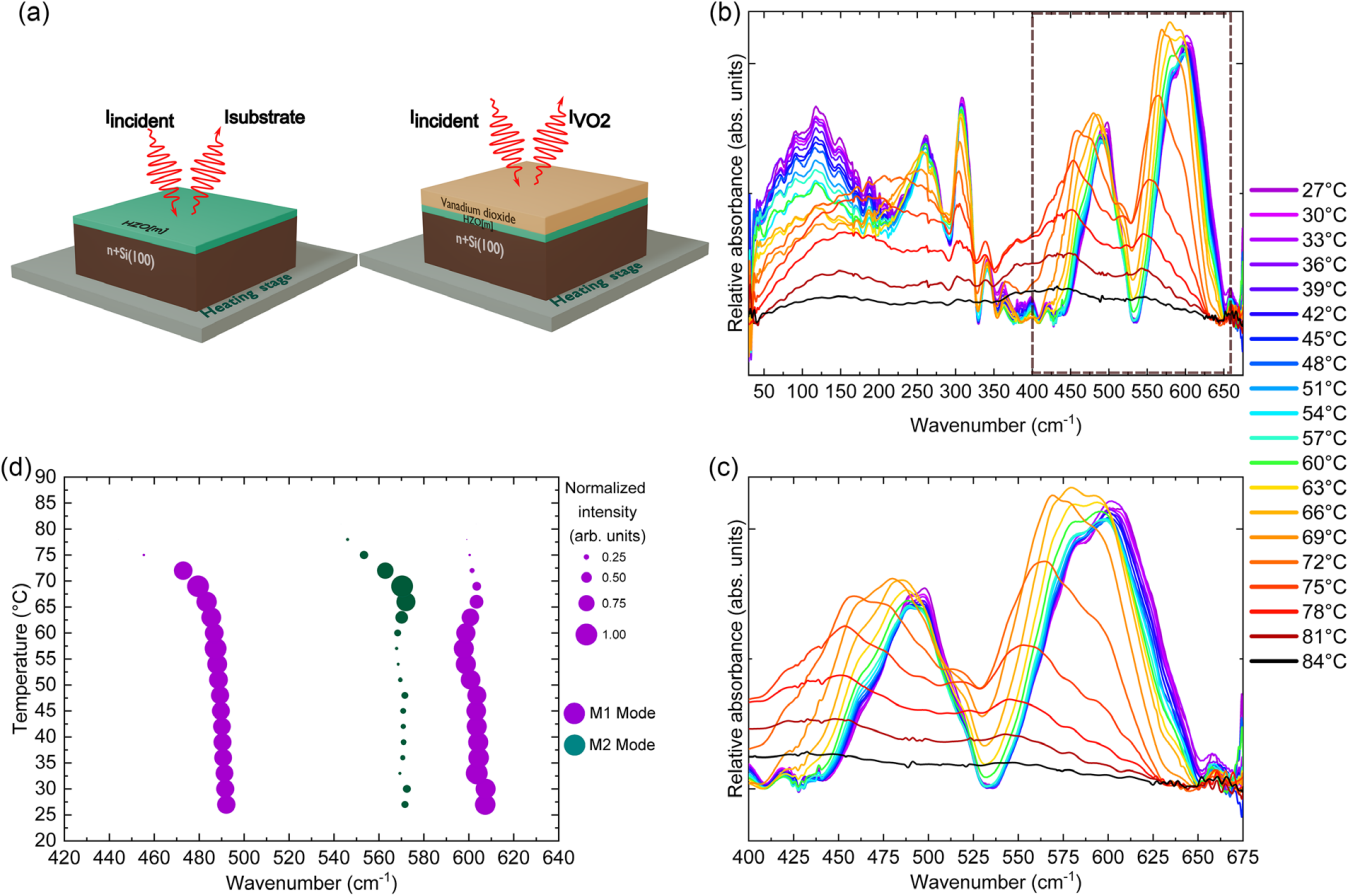
a) Schematic representation of reflectance measurements performed on thin films. *I*_substrate_ is the reference signal, i.e., from n^+^Si and HZO[m], and I_VO2_ is signal of the entire stack. b) Relative absorbance of VO_2_ on 700 °C_n^+^Si(100)/HZO[m] at various temperatures from 25 to 675 cm^−1^ illustrating emergence of M2 phonon mode in the vicinity of *T*_MIT_. c) Zoom on the 400–675 cm^−1^ region showing the appearance of the M2 phonon mode. d) Evolution of the M1 and M2 mode frequencies and intensities as a function of temperature.

Far‐IR (FIR) spectroscopy was performed from 30 to 650 cm^−1^ to investigate IR‐active phonons of VO_2_ in HZO[m]/VO_2_. **Figure** **6**a presents a scheme of reflectance measurements of the reference (*I*_substrate_), which is n^+^Si(100)/HZO[m], and that of the signal from VO_2_ and the reference ($I_{{\text{VO}}_{2}}$) at each defined temperature. Figure 6b shows the differential relative FIR absorption spectra for various temperatures (27–84 °C), in which one observes the measured phonon modes of VO_2_ on monoclinic HZO. The calculations for each spectrum are as follow:

(1) $$\text{Spectra}\left(T\right)=\log\left(\frac{I_{{\text{VO}}_{2}}\left(T\right)}{I_{\text{Substrate}}\left(T\right)}\right)-\log\left(\frac{I_{{\text{VO}}_{2}}\left(87{}^{\circ}C\right)}{I_{\text{Substrate}}\left(87{}^{\circ}C\right)}\right)$$

This treatment highlights the new phonon bands that evolve with temperature. At room temperature, we observed 10 out of the 15 IR‐active phonon modes (6 modes A_u_ and 4 B_u_ modes) predicted by the group theory. Our measurements agree with literature.^[^ ^65^, ^66^ ^]^ We clearly follow the predicted peaks at 260, 310, 340, 492, and 606 cm^−1^ as can be seen in our previous work.^[^ ^67^ ^]^

In Figure 6b, as the temperature increases, thermal expansion of the lattice leads to an apparent redshift in the phonon modes from 606 to 600 cm^−1^ and from 492 to 470 cm^−1^ at 72 °C. This motion can be attributed to the bending of the V—O—V associated to the M1 phase. Between 27 and 69 °C, there is a gradual change in the differential relative absorbance indicating the MIT. As we approach the *T*_MIT_, a phonon mode intensity around 570 cm^−1^ is seen to evolve in Figure 6c. This phonon frequency is ascribed to M2 phase which has been reported in Raman spectroscopy^[^ ^68^, ^69^ ^]^ at higher energy (650 cm^−1^). As a result of the redshift, the phonon mode of M2, which is anticipated to manifest around 650 cm^−1^, is observed at 570 cm^−1^ in our measurements, between 51 and 69 °C. The difference between M1 and M2 phonon modes arises from their inherent difference in V—O bonding. A_u_ mode of M1 phase at 606 cm^−1^ occurs due to the bending of the V—O—V octahedra while in the M2 phase, there are two different chains of V—O—V bonding resulting in a different energy of vibration.^[^ ^68^ ^]^ As can be seen in Figure 6d, the intensity of the M2 mode increases at 60 °C while the high energy intensity of the M1 mode starts to decrease. The possibility that this IR signature of the metastable phase originates from the T phase is discounted for the following reasons. The VO_2_ stress–temperature phase diagram^[^ ^70^ ^]^ informs that under tensile stress conditions, the sequence of phase transitions that usually occurs is M1 → T → M2 → R. The fact that the T phase precedes the M2 phase means that the appearance of a T phase would necessarily have to be followed by the passage through an M2 phase. This argument is further corroborated by the absence of a direct T—R phase boundary.^[^ ^69^ ^]^ Thus, if present, the triclinic phase would have always been detected along with the M2 phase. While the narrow temperature–stress band in which the T phase is expected could explain its absence in standard lab‐XRD setups, such a phase would be difficult to miss in synchrotron‐based IR spectroscopy, where enhanced resolution and sensitivity are achieved. Our detection of only one metastable phase through IR spectroscopy is thus attributed to the M2 phase, rather than the T phase. To our knowledge, it is the first direct observation of the transition M1—M2—R in VO_2_ in IR spectroscopy.

## 3 Conclusion

In conclusion, we have successfully integrated VO_2_ on silicon using CMOS compatible HZO (Hf_*x*_Zr_1−*x*_O_2_) buffer layers. This integration boasts a low hysteresis and fast thermal switching (low FWHM of $\frac{-\partial \log\rho }{\partial T}$), which is exciting for various applications such as sensors, nonvolatile optical memory, polarizer as well as electrical/optical switches where low power switching is desirable. On average, we demonstrate a hysteresis width of 4 °C and an FWHM of transition of 10 °C, which is significantly lower than previous works. These results show that HZO could be an ideal buffer layer as it does not allow the introduction of defects within VO_2_ during cyclic operations, which would circumvent the reliability issues. We note that the M2 phase of VO_2_ nucleates due to strain and contributes to the hysteresis. To our knowledge, the M1—M2—R transition in VO_2_ is reported using FIR spectroscopy for the first time. Specifically, we follow the apparition of the M2 mode and its disappearance with temperature. Our work presents a significant step toward integrating VO_2_ on silicon technology to exploit its versatility in various applications in next‐generation optoelectronics.

## 4 Experimental Section

4.1

4.1.1

##### Synthesis

The growth of the stack was carried out on 1 × 1 cm n^+^Si(100) substrates using magnetron sputtering (AC450 from Alliance Concept). Prior to the deposition process, the substrates were cleaned sequentially in an ultrasonic bath with acetone and ethanol, followed by the removal of native thermal oxide SiO_2_ on n^+^Si(100) using the well‐known buffered oxide etch process.^[^ ^71^ ^]^ A buffer layer of HZO was deposited on silicon at room temperature from a ceramic target of Hf_0.5_Zr_0.5_O_2_ using radio‐frequency sputtering as described in our previous work.^[^ ^72^, ^73^ ^]^ The deposited HZO varies in its Zr content as reported by our team before as a function of deposition pressure.^[^ ^28^ ^]^ The deposition was carried out at two different pressures, 5 × 10^−2^ mbar (HZO‐hp) and 5 × 10^−3^ mbar (HZO‐lp) to obtain a thickness of 13 and 16 nm, respectively. The n^+^Si(100)/HZO was then introduced into a rapid thermal annealing process at 600 °C for 30 s in an N_2_ atmosphere. This was followed by the deposition of VO_2_ from a V_2_O_5_ ceramic target by heating the substrates at 620 or 700 °C as referred in sample name. All thin films grown on silicon and *c*–sapphire at 700 °C were deposited simultaneously. The growth and annealing parameters determined for an optimized growth of VO_2_ on *c*–sapphire at a fixed magnetron power of 200 W are mentioned in **Table** **2**.

**Table 2:** HZO and VO_2_ thin‐film growth parameters.

Radio frequency (RF) magnetron sputtering		
Target–substrate distance	8 cm	
Base pressure [P]	5 × 10^−8^ < *P* < 5 × 10^−7^ mbar	
Deposited elements	Hf_0.57 ± 0.03_Zr_0.43 ± 0.03_O_1.7 ± 0.1_ Hf_0.54 ± 0.03_Zr_0.46 ± 0.03_O_1.8 ± 0.1_	VO_2_
Substrate	n^+^Si(100)	n^+^Si(100)/HZOa) *c*–sapphire (0001)
Target	Hf_0.5_Zr_0.5_O_2_	V_2_O_5_
Target RF power	100 W	200 W
Gas	Ar 50 sccm	Ar 50 sccm
Working pressure (mbar)	5 × 10^−2^	5 × 10^−3^
	5* × *10^−3^	
Substrate temperature	Room temperature	620 or 700 °C
Deposition speed [nm min^−1^]	4.3 (at 5 × 10^−2^ mbar)	3.4 for 620 °C and 4.2 for 700 °C
	8 (at 5 × 10^−3^ mbar)	

Rapid thermal annealing conditions of HZO		
Temperature [°C]	Atmosphere	Time [s]
600	N_2_	30

a) The name HZO is a class abbreviation for HZO‐hp and HZO‐lp. The compositions Hf_0.57 ± 0.03_Zr_0.43 ± 0.03_O_1.7 ± 0.1_ and Hf_0.54 ± 0.03_Zr_0.46 ± 0.03_O_1.8 ± 0.1_ are abbreviated as HZO‐hp and HZO‐lp, respectively. The terms “hp” and “lp” stand for high pressure (5 × 10^−2^ mbar) and low pressure (5 × 10^−3^ mbar).

##### Material Characterization

The thickness of each layer was obtained by X‐ray reflectometry (XRR). XRR and HR‐XRD measurements were carried out by a five‐circle goniometer SmartLab Rigaku diffractometer equipped with a 9 kW copper rotating anode. The films were characterized using specular (*θ*/2*θ*) as well as GIXRD technique. The GIXRD optical setup was made of a parabolic multilayer mirror for parallel beam setting, a Ni filter for Cu K*β* radiation, a parallel slit analyzer with a limit of resolution at 0.114°, and a 0D scintillating counter. The VO_2_ films were characterized using specular (*θ*/2*θ*) scan from room temperature to 85 °C to investigate the structural phase transition using a parallel beam with a two‐bounce Ge (220) monochromator.

XPS was employed to evaluate the electronic structure of the surface of VO_2_ thin films. The incident beam used was a monochromatic Al K*α* (1486.6 eV) and the spectrometer energy was calibrated to the Au 4*f*_7/2_ peak at 83.95 eV. All V 2*p*_1/2_ components were set at 50% of the integrated intensity of their corresponding 2*p*_3/2_ component to adhere to the theoretical electron occupancy rules. The pass energy setting of the analyzer yielded to an energy resolution of 0.04 eV. The spectral background due to rescattered electrons was subtracted using the Shirley method. Since the O 1*s* core level was close enough to the 2*p*_1/2_ level to have some influence, a unique Shirley background was subtracted. The measurements were recorded at room temperature and thus with VO_2_ in insulating phase. As no charging effect was detected, no electron gun flood was used, and the binding energy values were not charge corrected.

AFM was conducted in noncontact mode to investigate the effect of surface roughness and grain size distribution on MIT. The grain size of the VO_2_ films was quantitatively analyzed through the watershed method,^[^ ^74^, ^75^ ^]^ implemented using Gwyddion software.^[^ ^76^ ^]^

For VO_2_ grown on HZO buffer layers, infrared spectroscopy measurements as a function of temperature were performed in the FIR region in the reflectivity configuration on the IFS125MR Michelson interferometer at the infrared beamline advanced infrared line exploited for spectroscopy (AILES), Synchrotron SOLEIL. A 6 μm Mylar beamsplitter and a 4.2 K bolometer were used to perform measurement in the FIR range (30–650 cm^−1^) with the resolution of 2 cm^−1^. The optical setup allowed a strong focusing and a quasi‐normal incidence of the synchrotron beam onto the sample surface. In this configuration (infrared‐reflectivity‐absorption spectroscopy), the infrared beam was reflected by the substrate and then passed two times inside the sample. The resulting absorption spectra at each temperature were divided by the reference at the same temperature (sample with a thin layer of gold on the surface). We also subtracted the highest temperature (87 °C) for each spectrum. Then, baseline was removed using OPUS software. Interference fringes were removed using fringe removal on IGOR software and position of peaks were determined using gaussian peaks.

The electrical transport measurements of the samples were carried out using the four‐point probe measurement technique. We contacted four equally spaced, colinear probes to the film. The current was applied to the two outer tips and the voltage variation was measured between the inner two probes. The probes were mounted on springs to not damage the sample surface. The current value was ≈15 μA for all the samples. Before each run, current–voltage characteristics were measured to ensure that the system was not saturated by this current. A low bias current was used so that there was a negligible self‐heating effect. The temperature was controlled with a Pt100 thermistor, and the heating rate was set at 5 °C min^−1^.

HAADF imaging in STEM was performed in a Jeol JEM‐ARM200F NeoARM, equipped with a high‐brightness cold field emission gun, a CEOS ASCOR aberration corrector of the probe forming lenses, and operated at 80 kV. The cross‐section specimen for STEM analysis was prepared by focused‐ion‐beam milling. To calculate the *d–*spacing, an average value of diametrically opposite diffraction spots was considered. The *d–*spacing was calculated using first‐order reflection of Bragg's law. For all samples, the error in *d–*spacings was estimated from the FWHM of the diffraction spots; the maximum error was 0.01 Å.

## Conflict of Interest

The authors declare no conflict of interest.

## Author Contributions

**Swayam Prakash Sahoo**: Conceptualization (lead); Formal analysis (lead); Investigation (lead); Methodology (lead); Validation (lead); Visualization (lead); Writing—original draft (lead). **Matthieu Bugnet**: Investigation (equal); Resources (supporting); Software (equal); Supervision (supporting); Validation (equal); Visualization (equal); Writing—review & editing (equal). **Ingrid Cañero Infante**: Investigation (supporting); Methodology (supporting); Supervision (equal). **Victor Pierron**: Investigation (supporting). **Laurence Méchin**: Investigation (equal); Resources (supporting); Writing—review & editing (supporting). **Rebecca Cervasio**: Formal analysis (equal); Investigation (supporting); Resources (supporting); Software (supporting). **Pierre Hemme**: Formal analysis (supporting); Investigation (supporting). **Jean‐Blaise Brubach**: Investigation (equal); Methodology (equal); Resources (equal); Software (supporting); Validation (supporting); Visualization (supporting); Writing—review & editing (equal). **Pascale Roy**: Investigation (supporting); Methodology (supporting); Resources (equal); Software (supporting). **Luc G. Fréchette**: Funding acquisition (lead); Project administration (supporting); Supervision (supporting). **Anne D. Lamirand**: Project administration (supporting); Supervision (equal); Visualization (supporting); Writing—review & editing (equal). **Bertrand Vilquin**: Investigation (supporting); Project administration (equal); Resources (lead); Supervision (supporting); Validation (equal); Writing—review & editing (supporting).

## Supporting information

Supplementary Material

## Data Availability

The data that support the findings of this study are available in the supplementary material of this article.
