# Supplementary material for: Low Hysteresis Vanadium Dioxide Integrated on Silicon Using Complementary Metal‐Oxide Semiconductor Compatible Oxide Buffer Layer
Source: Small Sci. 2024 Oct 30;5(2):2400398. doi: 10.1002/smsc.202400398 (PMC11934903; doi:10.1002/smsc.202400398)
Supplement: Supplementary file 1 — Supplementary Material [file SMSC-5-2400398-s001.pdf]

Supporting Information

Low hysteresis vanadium dioxide integrated on silicon using CMOS compatible oxide buffer layer.

Swayam Prakash Sahoo, Matthieu Bugnet*, Ingrid Cañero Infante, Victor Pierron, Laurence Méchin, Rebecca Cervasio, Pierre Hemme, Jean-Blaise Brubach, Pascale Roy, Luc G. Fréchette, Anne D. Lamirand*, Bertrand Vilquin**

Mr. S.P. Sahoo, Prof. Dr. A.D. Lamirand, Prof. Dr. B. Vilquin
Université de Lyon, Ecole Centrale Lyon, INSA Lyon, CNRS, Institut des Nanotechnologies de Lyon (INL), UMR5270, 69130 Ecully, France
Email: swayam.prakash.sahoo@usherbrooke.ca
anne.lamirand@ec-lyon.fr; bertrand.vilquin@ec-lyon.fr

Dr. M. Bugnet
CNRS, INSA Lyon, UCBL, MATEIS, UMR 5510, Univ Lyon, Villeurbanne 69621, France
matthieu.bugnet@insa-lyon.fr

Dr. I.C. Infante
CNRS, INSA Lyon, Ecole Centrale Lyon, UCBL, CPE Lyon, INL, UMR5270, Univ Lyon, Villeurbanne 69621, France

V. Pierron, Prof. Dr. L. Méchin
Normandie Univ, UNICAEN, ENSICAEN, CNRS, GREYC (UMR 6072), 14000 Caen, France

Dr. R. Cervasio, Dr. P. Hemme, Dr. Jean-Blaise Brubach, Dr. P. Roy
Synchrotron SOLEIL, L'Orme des Merisiers, 91192 Gif-sur-Yvette Cedex, France

Mr. S.P. Sahoo, Prof. Dr. L. G. Fréchette
Laboratoire Nanotechnologies Nanosystèmes (LN2) - CNRS UMI-3463, Université de Sherbrooke, Sherbrooke, QC J1K 0A5, Canada
Institut Interdisciplinaire d'Innovation Technologique (3IT), Université de Sherbrooke, Sherbrooke, QC J1K 0A5, Canada

Contents

1. **Structural characterization**
X-ray reflectometry (XRR) of HZO thin films, and high-resolution X-ray diffraction (HRXRD) of VO_2 deposited on HZO, Si, and Al_2O_3.

2. **Electrical transport measurements**
Peak-fitting of $\frac{\partial\, lo}{\partial T}$ and tabulated data of MIT characteristics of VO_2 in different samples.

3. **X-ray photoelectron spectroscopy**
Evaluation of two methods of valency calculation namely, WPA and sensitivity factors, and correlating them to T_{MIT} and amplitude of transition.

4. **IR spectroscopy**
Plot of relative absorbance of VO_2 and tabulated data for fitting parameters of Lorentz oscillators.

1. Structural characterization

Figure S1a shows XRR measurements and fitting for HZO layers. The thicknesses of the HZO[m] and HZO[t] are 13 and 16 nm respectively. The fitting provided information on the roughness at different interfaces between the layers. **Figure S1b** reveals the specular(θ/2θ) high resolution XRD(HRXRD) of VO_2 measured between 2θ = 15° and 75°. This measurement shows the presence of VO_2[M1] phase and does not reveal any other polymorphic phase or stable oxide of V-O system. Si(200) forbidden peaks[1] occur at ≈ 32.98° with shoulders at ≈32.5°.

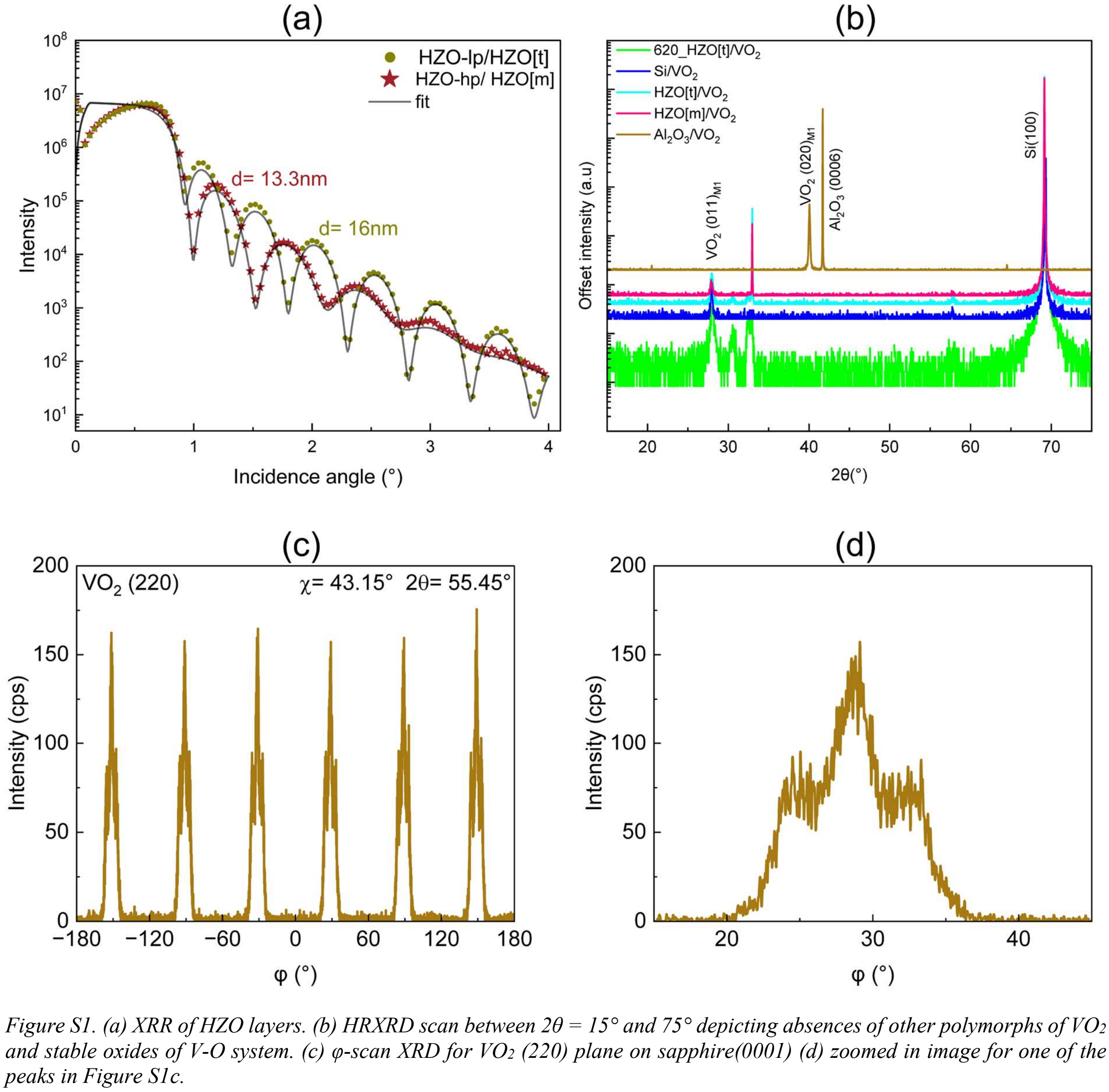

Figure S1. (a) XRR of HZO layers. (b) HRXRD scan between 2θ = 15° and 75° depicting absences of other polymorphs of VO_2 and stable oxides of V-O system. (c) φ-scan XRD for VO_2 (220) plane on sapphire(0001) (d) zoomed in image for one of the peaks in Figure S1c.

The (020) and (002) planes of monoclinic VO_2 have nearly identical interplanar spacings, leading to closely spaced 2θ values. To differentiate between these planes, a φ-scan XRD

analysis was carried out on the (220) reflection (2θ = 55.45°) of monoclinic VO_2, which forms χ-angles of 43.14° with the (020) plane and 68.35° with the (002) plane [2].

Figure S1c shows off-axis φ-scan performed at 2θ = 55.45° and χ = 43.14°. The measurement (not shown here) at 2θ = 55.45° and χ = 68.35° did not show any peaks confirming the absence of (002) peaks. The VO_2 film grown epitaxially on the c-plane of the Al_2O_3 substrate therefore exhibits a pronounced (020) orientation, with the in-plane crystal lattice showing pseudo-six-fold symmetry. Notably, each of the six primary peaks is flanked by two additional satellite peaks which is shown in **Figure S1d** for one of the primary peaks. This six-fold symmetry results[3] from a triple-domain structure that remains consistent under 60° rotations in the (020) basal plane. The presence of satellite peaks is accounted for by the angular mismatch between the β angle of VO_2 (122.6°) and the γ angle of Al_2O_3 (120°) within the triple-domain structure.

2. Electrical transport measurements

Figure S2 shows the deconvolution of $\frac{-\partial \log}{\partial T}$ into two peaks for heating as well as cooling half-cycle using pseudo-Voigt functions with predominantly Gaussian nature. We also note that Gaussian fitting has been used in previous works[4,5] to fit $\frac{-\partial \mathrm{lo}}{\partial T}$ data despite there being no theoretical reasoning on the shape of the peak profile. For our data, pure Gaussian functions did not result in a good fitting. The profile shape factor used for each peak within the square brackets in Table S1. As illustrated in the sample Si/VO_2, these peaks depict M1-M2 and M2-R transition. From this deconvolution, the hysteresis widths ΔT_H of M1-M2 and M2-R phase transitions are calculated from the difference of maxima of $\frac{-\partial \log \rho}{\partial T}$ for corresponding transitions. %M2 phase is the percentage of total domains undergoing M1-M2 transition in each half-cycle and is defined as the normalized area under $\frac{-\partial \log}{\partial T}$ in each half-cycle. These data are tabulated in **Table S1.**

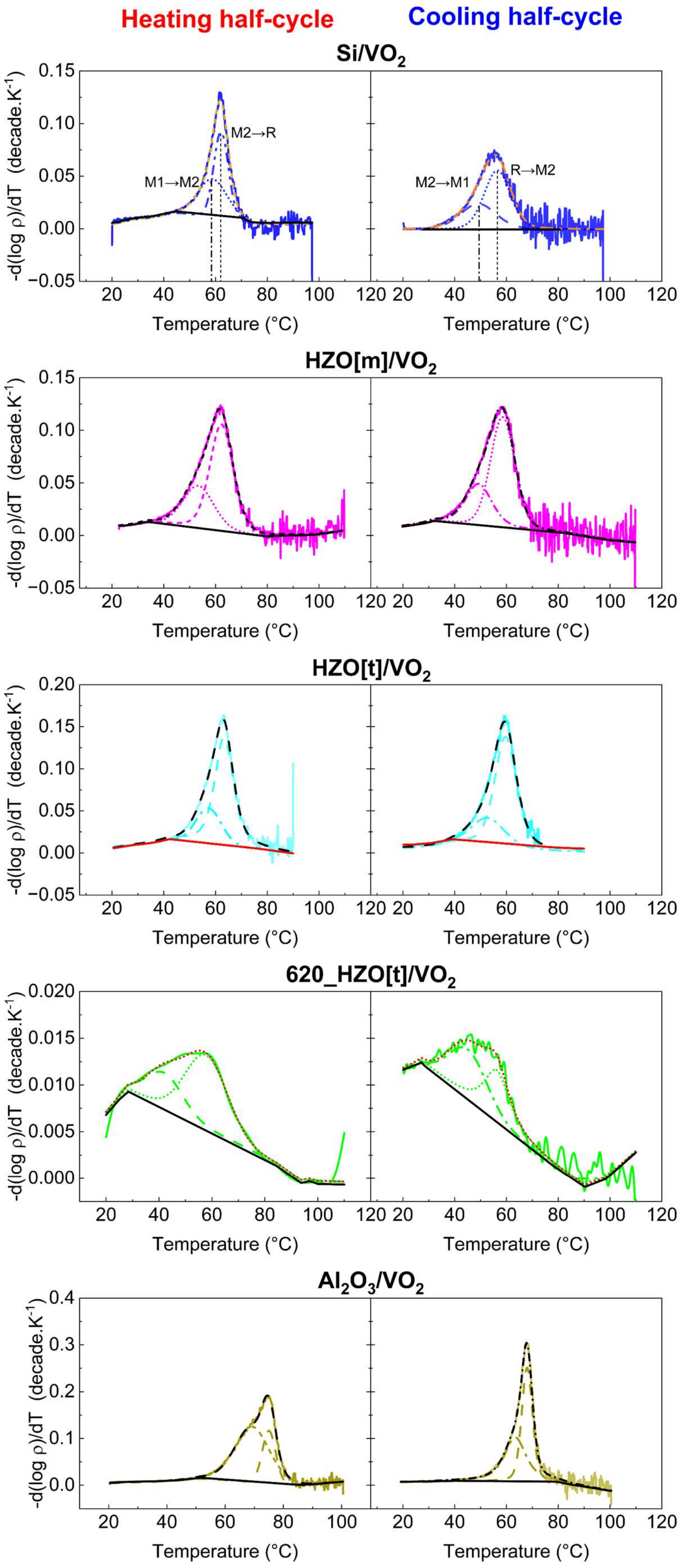

Figure S2. Peak- fitting of $\frac{-\partial\, lo}{\partial T}$ *curve for heating and cooling cycle to quantify the M2 phase*

The weighted FWHM of transition presented in **Figure 4e** and **Figure 5b** corresponds to the mean value of the two FWHMs $\digamma$ (in each half-cycle; attributed to M1-M2 and M2-R transition) weighted by the area of their peak (a). Mathematically,

$$weighted\ FWHM = \frac{a_{M1\rightarrow M2} * \digamma_{M1\rightarrow M2} + a_{M2\rightarrow R} * \digamma_{M2\rightarrow R}}{100}$$

Table S1. MIT characteristics of samples. Data within the square brackets indicate peak shape factor, where the scale spans from 0 to 1 with 0 being pure Gaussian profile and 1 representing pure Lorentzian and any value in between is the linear additive combination of both.

**Sample**	**Transition temperature (°C)**						**Hysteresis ΔT_H (°C)**		**Amplitude of the MIT (in decade)**
	**Heating cycle (T_h)**			**Cooling cycle (T_c)**			$M1\leftrightarrow M2$	$M2\leftrightarrow R$	
	$M1\rightarrow M2$	$M2\rightarrow R$	*%M2*	$R\rightarrow M2$	$M2\rightarrow M1$	*%M2*			
620_HZO[t]/VO_2	42.1 [0]	58.0 [0]	28.6	56.4 [0.6]	45.2 [0]	52.8	3.1	1.6	0.22
HZO[t]/VO_2	57.5 [0]	63.5 [0.5]	26.5	60.1 [0.5]	53.6 [0.2]	25.7	3.9	3.4	2.03
HZO[m]/VO_2	53.5 [0.2]	62.5 [0.34]	33.6	58.6 [0.45]	49.4 [0.18]	30.4	4.1	3.9	1.93
Si/VO_2	55.0 [0]	62.1 [0]	46.8	56.6 [0.25]	49.2 [0.3]	37.3	5.8	5.5	1.1
Al_2O_3/VO_2	69.3 [0.28]	75.2 [0]	74.5	68.3 [0.38]	64.8 [0.6]	47.3	4.5	6.9	3.02

3. X-ray photoelectron spectroscopy

The valency in XPS can be calculated using two approaches. Firstly, by calculating the area normalized by sensitivity factors, and secondly, by employing the weight percent average (WPA) of V$2p_{3/2}$ peaks, as documented in the literature[6].

WPA (weight percent average) method is given by:

$$Valency(WPA\ method) = \frac{a_{V^{4+}} * 4 + a_{V^{5+}} * 5}{100}$$

where, a is the percentage area of V^{4+} and V^{5+} under the $V2p_{3/2}$ peak. As presented in **Figure S2,** we observe that the trends of the amplitude of transition and the variation of T_{MIT} with valency (WPA method) are incorrect as higher oxygen vacancies lower the T_{MIT} and the amplitude of transition.

Valency calculation using sensitivity factors unlike the WPA method utilizes the O *1s* peak and the respective sensitivity factors (a combination of cross-section of interaction and their inelastic mean-free paths). We label this method as valency (sensitivity factor) and is mathematically given by:

$$Valency(sensitivity\ factors) = \frac{2.\frac{I^{O\ 1s}}{S^{O\ 1s}}}{\frac{I^{V\ 2p_{3/2}}}{S^{V\ 2p_{3/2}}}}$$

Where I and S are intensities and sensitivity factors of respective species.

XPS measurements were correlated to MIT characteristics from electrical characterization, namely the amplitude of transition and the variation of T_{MIT}. **Figure S3** showcases their trend for both approaches of calculating valencies. As it is known that oxygen vacancies reduce the T_{MIT} to lower temperature and also reduces the amplitude of transition, valency (sensitivity factor) method perfectly correlates to MIT characteristics and therefore is our chosen method of correlating to FWHM of transition. As can be seen valency(sensitivity factors) results in valency less than 4 while WPA method provides a valency higher than 4. Furthermore, we observe low relative absorbance(ΔA) in room temperature IR spectroscopy measurements in **Figure S4** (@ 309 cm^{-1}, ΔA = 0.17 and @ 606 cm^{-1}, ΔA = 0.10). This indicates oxygen deficient nature of VO_2 in the bulk of the thin film, thereby also confirming that the presence of V^{5+} denotes the surface oxidation of VO_2 thin film.

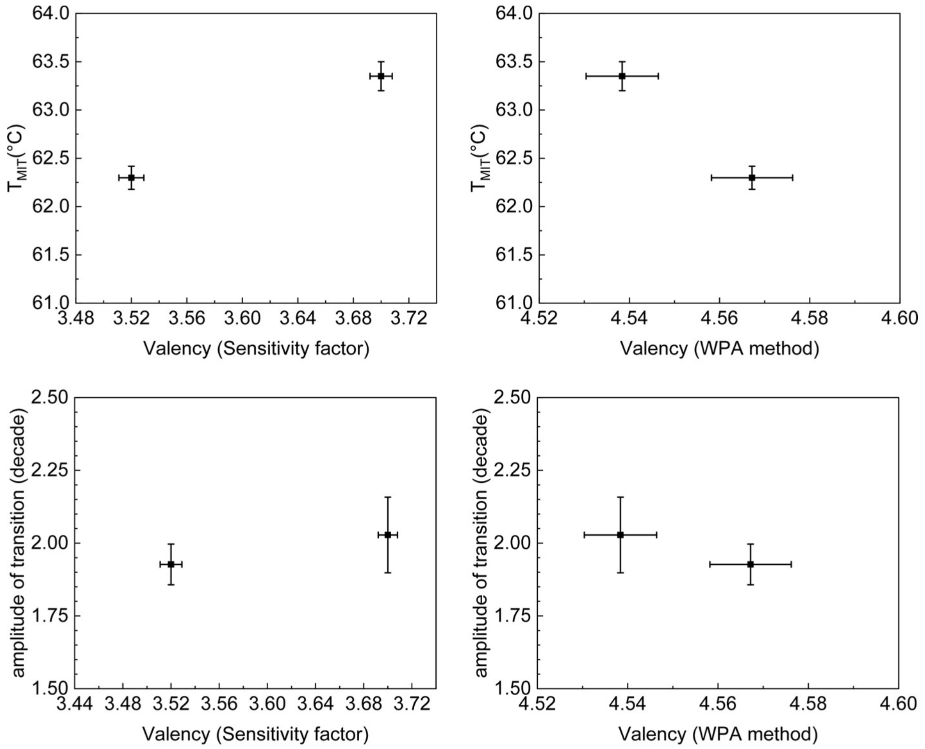

Figure S3. Evaluation of two methods of valency calculation namely, WPA and sensitivity factors, and correlating them to T_{MIT} and amplitude of transition.

4. IR spectroscopy

The experimental data of relative reflectance was modeled using Kramers-Kronig constrained Drude Lorentzian (DL)damped oscillators to obtain the dielectric-function parameters using RefFIT software[7]. Relative Reflectance for various temperatures across the T_{MIT} were modeled. The DL function we used is in the form[8,9]

$$\varepsilon(\omega) = \varepsilon_\infty + \sum_i \frac{\omega_p^2}{\omega_T^2 - \omega^2 - i\gamma_i\omega}$$

Where, $\omega_p, \omega_T\ and\ \gamma_i$ are the plasma frequency, transverse optical frequency (*or binding frequency associated a restoring force acting on bound charged particles towards their equilibrium position*), and scattering rate (damping force) of the ith DL oscillator.

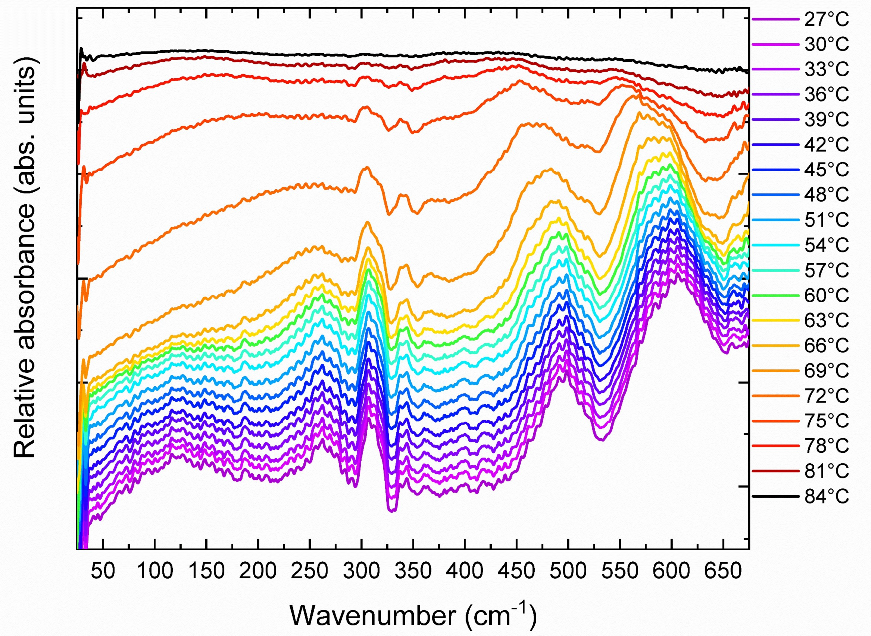

Figure S4. The plot of relative absorbance of VO_2 showcasing reducing absorbance with increasing temperature as a result of transitioning into metallic phase.

Table S2. Fitting parameters of Lorentz oscillators for (i) insulating monoclinic (27°C) and (ii) metallic (78°C) rutile VO_2.

(i)

**A_u mode**			**B_u mode**	
ω_T (cm^{-1})	ω_p(cm^{-1})		ω_T(cm^{-1})	ω_p(cm^{-1})
189	1839.64		279	25.08
285	1104.24		327	834.67
310	33.66	ε_∞= 11	349	1648.15
333	1055.37		377	2041.03
515	1218.74			
604	1591.13			

(ii)

ω_T (cm^{-1})
286
386
406
436
478
582

References:

[1] P. Zaumseil, *J Appl Crystallogr* **2015**, *48*, 528.
[2] M. R. Bayati, R. Molaei, F. Wu, J. D. Budai, Y. Liu, R. J. Narayan, J. Narayan, *Acta Materialia* **2013**, *61*, 7805.
[3] L. L. Fan, Y. F. Wu, C. Si, G. Q. Pan, C. W. Zou, Z. Y. Wu, *Applied Physics Letters* **2013**, *102*, 011604.
[4] Y. Ji, Y. Zhang, M. Gao, Z. Yuan, Y. Xia, C. Jin, B. Tao, C. Chen, Q. Jia, Y. Lin, *Sci Rep* **2014**, *4*, 4854.
[5] A. Boontan, E. K. Barimah, P. Steenson, G. Jose, *ACS Appl. Mater. Interfaces* **2023**, *15*, 51606.
[6] G. Silversmit, D. Depla, H. Poelman, G. B. Marin, R. De Gryse, *Journal of Electron Spectroscopy and Related Phenomena* **2004**, *135*, 167.
[7] A. B. Kuzmenko, *Review of Scientific Instruments* **2005**, *76*, 083108.
[8] J. D. Jackson, *Classical electrodynamics*, Wiley, New York, **1999**.
[9] I. Ben Soltane, F. Dierick, B. Stout, N. Bonod, *Advanced Optical Materials* **2024**, 2400093.
